# Supplementary material for: The impact of COVID-19 in children with Sickle Cell Disease: Results of a multicentric registry
Source: PLoS One. 2023 Apr 6;18(4):e0282423. doi: 10.1371/journal.pone.0282423 (PMC10079108; doi:10.1371/journal.pone.0282423)
Supplement: Attachment — Submitted filename: Response to reviewers.docx [file pone.0282423.s001.docx]

Porto Alegre, September 20th 2022

Dear Emily Chenette

Editor in Chief

Plos One

Dear Emily Chenette

First of all we would like to thank the reviewers for the time dedicated and the opportunity of answering these questions and improving our paper. They were very relevant to qualify the information we wanted to show. I approached each of their concerns very carefully.

Please find here below our answers and some supplementary data.

Changes requested by reviewers that should be added to the article were entered in the file and are highlighted in yellow.

Reviewer #1: The authors present the short-term outcomes of pediatric patients with Sickle Cell Anemia who had symptoms of Covid-19 prior to vaccination. Data were obtained from more than one treatment center in Brazil, located in the Midwest, Southeast and South regions. The results were properly analyzed and discussed, with objectivity.

Suggestion to authors: remove lines 2-9, page 8, from the Introduction, as they contain information that is already well known and unnecessary considering the profile of the journal's readers.

*Thank you very much for your attentive review. We removed this part from the text as suggested.*

The paragraph between lines 13-19 is important for contextualization and could be developed.

*Thanks for the suggestion. As the other reviewer also requested we have added more information on the pathophysiology of the disease prior to this paragraph.*

In the same way, line 66, page 10, Results, must be deleted as it is already found in Method.

*We removed this part as suggested.*

Having made these considerations, the manuscript is ready for publication.

Reviewer #2: The main objective of the manuscript is to describe the behavior and evolution of sickle cell disease patients who had real-time PCR test positive for SARS-CoV2. The study included patients from Central and Southeastern regions of Brazil, late the authors include the South, and it needs to be included in the abstract, and presents data for one year since the diagnosis of COVID-19 was realized.

General concept comments

The manuscript covers a field that still have little knowledge of, that is the COVID-19; and when it comes to COVID-19 in sickle cell disease, this scarcity of data is even greater, given the complexity of the two diseases. In this way, the focus of the work is important and pertinent. What I consider an area of weakness in the manuscript is really the number of individuals included in the study, 25 in total, 20 with sickle cell anemia and 5 with SC disease, a very small number for the conclusions presented by the authors. However, this reviewer will make the considerations to assist in the general conception of the analysis of the manuscript.

In addition, this reviewer believe that it is important to discuss a little more about the pathophysiology and repercussions of sickle cell disease, both in the introduction and in the discussion, especially in the latter, since the most clinical results found were not discussed, which in my view do not match the conclusion placed by the authors that “In conclusion, COVID-19 in children with SCA did not lead to an increase in mortality rates, unlike other respiratory viral infections known for causing serious complications in these patients”, since the number of patients followed up does not allow the authors to reach such a conclusion.

*Thank you for your comment.*

*We adjusted the text and included information on the pathophysiology and repercussions as suggested. We understand that it will indeed make the text more informative and robust.*

*The conclusion was corrected and now it is written:*

*“In conclusion, in our sample of children with SCA, COVID-19 did not lead to an increase in mortality rates. This result appears to be different from that previously described in other respiratory viral infections known to cause serious complications in these patients.”*

Firstly, there is a mistake in the use of the term sickle cell disease, as the series comprises individuals with sickle cell anemia (HbSS) and SC disease (HbSC), the authors should change the general term to sickle cell disease, including both groups investigated. This change must also occur in the title and keywords, which must be changed to sickle cell disease.

*We made this change, thanks for the suggestion.*

The authors describe the symptoms presented by patients who have a very diverse clinical picture, some classified as moderate to severe. The authors also refer to data loss and data entry, which may have caused some bias in the study. Considering that the number of patients analyzed is extremely small, it will be important to be very careful with the conclusions.

a. Another important aspect is related to the previous data of these patients; did they have many symptoms before COVID? What about laboratory tests?

*We don't have this information. All the patients were followed up at the reference centers cited.*

b. What were the units of each variable presented on table 1?

*Units were included in the table as suggested.*

c. Were the laboratory methods similar in all institutions participating in the study?

*The limits of normal results were compared and standardized between centers .*

d. Since the study took a year to collect data, do you hear a follow-up of patients? Today we know that long-term and post-COVID-19 are reality, how did these patients maintain themselves during the study period?

*Your comment is very pertinent and responds to a wish from our team as well. We are currently seeking financial support that would allow us to monitor these patients and eventually extend this cohort to other centers in the country.*

It is these questions that demonstrate that the study, despite being well structured, still has questions to be presented, providing a clear overview of who these patients are and how they evolved with the development of the disease. Another aspect is the comparison between laboratory data between SS and SC individuals with COVID, is SC expected to be less severe? This can be an interesting question, but it needs a more robust number of individuals to be well answered.

The authors agree with the reviewer on this point. There follows a very interesting question to be answered in a follow-up or expansion of the present study.

Were these patients previously treated with hydroxyurea? Could this make a difference in the response to SARS-CoV-2 infection?

*We had 12 patients using hydroxyurea. This current n value does not allow comparison of groups with and without hydroxyurea. The idea is quite interesting and could be included in the follow-up of the study. The authors thank the reviewer for the comment and reflection.*

Please explain the study design, what it means “a multicenter observational cohort study was conducted on prospective data and retrospective analyses”, it was not clear.

*We corrected this sentence in the abstract to :*

*A multicenter prospective study was conducted in five hematological centers from Central and Southeast Brazil, starting in April 2020.*

Regarding the table presented in the manuscript, the table 1, it will be important that some numbers be revised, as they are not currently presented in English.

*We regret this act of inattention. These numbers have been properly corrected.*
