# Supplementary material for: The impact of COVID-19 in children with Sickle Cell Disease: Results of a multicentric registry
Source: PLoS One. 2023 Apr 6;18(4):e0282423. doi: 10.1371/journal.pone.0282423 (PMC10079108; doi:10.1371/journal.pone.0282423)
Supplement: Attachment — Submitted filename: response to reviewers.docx [file pone.0282423.s002.docx]

Porto Alegre, January 17th 2023

Dear Emily Chenette

Editor in Chief

Plos One

Dear Emily Chenette

First of all we would like to thank the reviewers for the time dedicated and the opportunity of answering these questions and improving our paper. They were very relevant to qualify the information we wanted to show. I approached each of their concerns very carefully.

Please find here below our answers and some supplementary data.

Changes requested by reviewers that should be added to the article were entered in the file and are highlighted in yellow.

Reviewer #1: The authors have adequately addressed all the comments raised in the previous round of review and this manuscript is acceptable for publication

*Thank you very much for your attentive review*

Reviewer #2: The authors developed a careful review of the manuscript and emphasized aspects that this reviewer considered relevant. The authors initially included a first paragraph about sickle cell disease, which complements the manuscript and supports the study that was developed.

As stated in the analysis carried out earlier, this reviewer considers that the data presented provide important information about covid-19 in sickle cell disease, reinforcing the need for additional studies aimed at learning more about this interaction. However, this reviewer still felt the need for some corrections in the text, as well as a grammar review so that the manuscript is uniform in terms of the choice of English language.

Regarding to the manuscript, I noticed that there is a need for some adjustments throughout the text.

1- It will be important that in the first citation of sickle cell disease, the author already includes the acronym SCD and already includes it in the text from that moment.

*Thank you, we corrected the missing abbreviations.*

2- In the first paragraph the authors still in the first paragraph of the introduction section, in line 11 in the text "It is estimated that 1,000 children are born with it in Brazil annually", please replace it with "children are born with SCD", it is important to mention it in the sentence and not leave as a hidden subject.

*Thank you, we corrected the sentence to "It is estimated that 1,000 children are born with SCD in Brazil annually”.*

3- line 14, first paragraph of the introduction, replace abnormal hemoglobin with variant hemoglobin.

*Thank you, we replaced the word “abnormal”.*

4- in general, the text mixes American English with British English, authors should choose one of the two and keep the language constant in the text.

*Thank you, we corrected the English and we highlighted all the changes made.*

5- In the session called statistical analysis, should be used for description of normally and non-normally distributed data, and not asymmetric distribution.

*Thank you, we replaced the word " asymmetric distribution".*

6- Lines 72-73, use the same description mentioned before, the authors do not need to repeat, use HbSS and HbSC.

*Thank you, we corrected the sentence to " Fetal hemoglobin (HbF) levels were significantly higher in patients with Hb SS".*

7- in the text include in line 123, the authors mention that studies have shown about the predisposition of organs to hypoxia, but only include a single reference, it will be important to mention others or adjust the sentence for that single reference.

*Thank you, we included other references.*
